# Impact of solitary pulmonary nodule size on qualitative and quantitative assessment using 18F-fluorodeoxyglucose PET/CT: the SPUTNIK trial

**DOI:** 10.1007/s00259-020-05089-y

**Published:** 2020-11-01

**Authors:** J. R. Weir-McCall, S. Harris, K. A. Miles, N. R. Qureshi, R. C. Rintoul, S. Dizdarevic, L. Pike, Heok K. Cheow, Fiona J. Gilbert, on behalf of the SPUtNIk investigators, Anindo Banerjee, Lucy Brindle, Matthew Callister, Andrew Clegg, Andrew Cook, Kelly Cozens, Philip Crosbie, Sabina Dizdarevic, Rosemary Eaton, Kathrin Eichhorst, Anthony Frew, Fergus Gleeson, Ashley Groves, Sai Han, Jeremy Jones, Osie Kankam, Kavitasagary Karunasaagarar, Lutfi Kurban, Louisa Little, Jackie Madden, Clare McClement, Ken Miles, Patricia Moate, Charles Peebles, Lucy Pike, Fat-Wui Poon, Donald Sinclair, Andrew Shah, Luke Vale, Steve George, Richard Riley, Andrea Lodge, John Buscombe, Theresa Green, Amanda Stone, Neal Navani, Robert Shortman, Gabriella Azzopardi, Sarah Doffman, Janice Bush, Jane Lyttle, Kenneth Jacob, Joris van der Horst, Joseph Sarvesvaran, Barbara McLaren, Lesley Gomersall, Ravi Sharma, Kathleen Collie, Steve O’Hickey, Jayne Tyler, Sue King, John O’Brien, Rajiv Srivastava, Hugh Lloyd-Jones, Sandra Beech, Andrew Scarsbrook, Victoria Ashford-Turner, Elaine Smith, Susan Mbale, Nick Adams, Gail Pottinger

**Affiliations:** 1 Department of Radiology, Biomedical Research Centre, University of Cambridge School of Clinical Medicine, University of Cambridge, Cambridge, CB2 0QQ UK; 2 Department of Radiology, Royal Papworth Hospital, Cambridge, UK; 3 Public Health Sciences and Medical Statistics, University of Southampton, Southampton, UK; 4 Institute of Nuclear Medicine, University College London, London, UK; 5 Department of Thoracic Oncology, Royal Papworth Hospital / Department of Oncology, University of Cambridge, Cambridge, UK; 6 Departments of Imaging and Nuclear Medicine and Respiratory Medicine, Brighton and Sussex University Hospitals NHS Trust, Brighton and Sussex Medical School, Brighton, UK; 7 King’s College London and Guy’s & St Thomas’ PET Centre, School of Biomedical Engineering and Imaging Sciences, King’s College London, London, UK; 8 Addenbrookes Hospital, Cambridge University Hospitals NHS Trust, Cambridge, UK

**Keywords:** Solitary pulmonary nodule (SPN), DCE-CT, PET/CT, Diagnostic imaging, Lung cancer, Diagnostic accuracy trial, Cost-effectiveness

## Abstract

**Purpose:**

To compare qualitative and semi-quantitative PET/CT criteria, and the impact of nodule size on the diagnosis of solitary pulmonary nodules in a prospective multicentre trial.

**Methods:**

Patients with an SPN on CT ≥ 8 and ≤ 30 mm were recruited to the SPUTNIK trial at 16 sites accredited by the UK PET Core Lab. Qualitative assessment used a five-point ordinal PET-grade compared to the mediastinal blood pool, and a combined PET/CT grade using the CT features. Semi-quantitative measures included SUVmax of the nodule, and as an uptake ratio to the mediastinal blood pool (SUR_BLOOD_) or liver (SUR_LIVER_). The endpoints were diagnosis of lung cancer via biopsy/histology or completion of 2-year follow-up. Impact of nodule size was analysed by comparison between nodule size tertiles.

**Results:**

Three hundred fifty-five participants completed PET/CT and 2-year follow-up, with 59% (209/355) malignant nodules. The AUCs of the three techniques were SUVmax 0.87 (95% CI 0.83;0.91); SUR_BLOOD_ 0.87 (95% CI 0.83; 0.91, *p* = 0.30 versus SUVmax); and SUR_LIVER_ 0.87 (95% CI 0.83; 0.91, *p* = 0.09 vs. SUVmax). The AUCs for all techniques remained stable across size tertiles (*p* > 0.1 for difference), although the optimal diagnostic threshold varied by size. For nodules < 12 mm, an SUVmax of 1.75 or visual uptake equal to the mediastinum yielded the highest accuracy. For nodules > 16 mm, an SUVmax ≥ 3.6 or visual PET uptake greater than the mediastinum was the most accurate.

**Conclusion:**

In this multicentre trial, SUVmax was the most accurate technique for the diagnosis of solitary pulmonary nodules. Diagnostic thresholds should be altered according to nodule size.

**Trial registration:**

ISRCTN - ISRCTN30784948. ClinicalTrials.gov - NCT02013063

**Supplementary Information:**

The online version contains supplementary material available at 10.1007/s00259-020-05089-y.

## Introduction

Solitary pulmonary nodules, defined as distinct focal pulmonary lesions ≤ 30 mm, are a relatively common finding on chest CT and present a significant opportunity to improve patient outcomes as early diagnosis of lung cancer results in excellent survival rates following surgical resection [1]. However, not all SPNs are due to lung cancer with rates of malignancy ranging from 3% in screening detected nodules to 60% in clinically detected nodules [2–5]. The accurate characterisation of SPNs is an on-going diagnostic challenge with significant associated health costs [6].

The probability of malignancy of a nodule is strongly related to its size, ranging from 6% in nodules 5–10 mm to 64% in nodules greater than 20 mm in size [7]. As a result, downstream work-up of a nodule is dependent on its size with nodules < 8mms requiring CT follow-up, and nodules 8–30 mm requiring further work-up with biopsy or PET/CT [8]. ^18^Fluorine fluorodeoxyglucose positron emission tomography/computed tomography (PET/CT) has a high accuracy for the diagnosis of malignancy in nodules with a recent meta-analysis reporting a sensitivity of 89% and a specificity of 70% [5]. All the studies included in this meta-analysis used SUVmax cutoffs to determine malignancy status. However, SUV is a relative measure of FDG uptake, which is prone to variability as a result of scanner features, patient factors, imaging protocols and reconstruction algorithms [9]. Due to this limitation, several authors have suggested the use of a visual ordinal scale comparing the nodule uptake to that of the mediastinal blood pool [10]. Normalising the tumour SUVmax to background tissue such as the blood pool or liver to create a standardised uptake ratio (SUR) can significantly improve the variability in quantification of tumour uptake and may overcome some of the inherent limitations of isolated tumoral SUVmax measurement [11]. This in turn may allow for a more nuanced approach to nodule assessment and follow-up through the use of a semi-quantitative metric rather than allowed for within the visual ordinal system.

The purpose of the current study was to compare the diagnostic accuracy of qualitative and semi-quantitative metrics of PET/CT in pulmonary nodules in a prospective, multicentre multivendor environment.

## Methods

This is a pre-specified secondary analysis of the SPUTNIK (Accuracy and Cost-Effectiveness of Dynamic Contrast Enhanced Computed Tomography in the Characterisation of Solitary Pulmonary Nodules) trial. This prospective multicentre observational study compared the diagnostic accuracy of PET/CT with that of dynamic contrast enhanced CT in a cohort of patients with an indeterminate solitary pulmonary nodule (SPN) (Trial registration: ISRCTN - ISRCTN30784948; ClinicalTrials.gov - NCT02013063). The full trial protocol has been previously published [12]. The SPUtNIk Trial was approved by the South West Research Ethics Committee Centre (UK). All participants provided written informed consent.

### Settings and participants

Participants were recruited from secondary or tertiary outpatient settings at 16 hospitals within the UK.

The inclusion criteria were as follows: soft tissue solitary indeterminate pulmonary nodule of ≥ 8 mm and ≤ 30 mm on axial plane measured on lung window using conventional CT scan with no evidence strongly indicative of malignancy (such as metastases or local invasion).

Exclusion criteria were as follows: pregnancy; history of malignancy within the past 2 years; confirmed aetiology of the nodule at the time of qualifying CT scan; biopsy of nodule prior to the PET/CT; contra-indication to potential radiotherapy or surgery; contraindication to scans (assessed by local procedures).

#### PET/CT acquisition

The PET/CT examinations were performed on 25 scanners at the 16 participating centres. Patients were fasted for 6 h (4 h for diabetics) before receiving the injection of ^18^F-FDG, with instructions to avoid strenuous exercise for 6 h before the scan. Blood glucose levels were checked to ensure they were less than 11 mmol/l prior to injection. A low-dose CT without contrast was acquired before the PET acquisition for attenuation correction of the PET images. The injected dose of ^18^F-FDG is dependent on the PET system used and the patient weight according to local protocols. Whole-body imaging began at 60 ± 10 min (mean ± SD) after injection and was acquired from the skull base to the level of the mid-thigh and reconstructed using attenuation correction.

All scanners underwent baseline accreditation and annual quality assurance testing by the UK PET Core Lab [13]. In addition, the UK PET core lab performed technical checks and image quality review of all studies.

#### PET/CT analysis

All PET/CT examinations were reported by local trained physicians prior to biopsy using visual grading and SUVmax of the nodule. For this substudy, all images were re-read by a central lab blinded to the clinical details and nodule status by a single reader (JRWM) using a dedicated PET/CT reporting platform (ADW 4.4, GE) using attenuation corrected images. For the qualitative analysis, the CT and PET images were graded. The CT features were graded as follows: 0 = round, well-defined lesion with laminated or popcorn calcification; 1 = inflammatory features, e.g., air bronchograms, enfolded lung; 2 = smooth well-defined margins, uniform density; 3 = lobulated, spiculated or irregular margins; 4 = evidence of metastases. The PET features were graded as follows: 0 = no visible uptake; 1 = uptake less than mediastinal blood pool; 2 = uptake comparable to mediastinal blood pool; 3 = uptake greater than mediastinal blood pool; 4 = evidence of distant metastases (i.e., M1 disease). For the semi-quantitative analysis, the SUVmax and mean of the nodule were measured, as were the SUVmax and mean of the ascending aorta at the level of the arch, and within the right lobe of the liver. SUV ratios (SUR) were calculated between the nodule SUVmax, and the mediastinal blood pool (SUR_BLOOD_) and liver (SUR_LIVER_).

Pre-specified criteria for malignancy for each of the metrics were as follows:

- Semi-quantitative: SUVmax ≥ 2.5; SUR_BLOOD_ ≥ 1.56; SUR_LIVER_ ≥ 1.12 [14]
- Qualitative: PET grade ≥ 3;
- Combined PET/CT: Grade 4 on PET or CT, Grade 3 on PET and ≥ Grade 2 on CT, or Grade 2 on PET and ≥ Grade 3 on CT [15, 16]

Further exploratory analyses were also performed using optimised thresholds from the ROC curves derived in the current study.

### Reference standard

For the diagnostic accuracy, the reference standard was histology or completion of 2 years of nodule surveillance. For a nodule to be diagnosed as malignant, histological confirmation was required or an increase in nodule size with a specialist thoracic oncology multi-disciplinary team (MDT) consensus of certainty of malignancy where biopsy/resection was not possible. Benign status could be established through either histology, or through demonstration of stability over 2 years of CT monitoring.

### Statistical analysis

Continuous data is presented as mean ± SD, while ordinal data is provided as *N* (%). We considered the diagnostic accuracy of qualitative and semi-quantitative PET/CT in relation to a diagnosis of lung cancer by 2 years. The diagnostic accuracy of the tests was assessed by sensitivity, specificity, positive predictive value negative predictive value and overall accuracy for the pre-specified end points with results for each of these reported with 95% CIs. Further exploratory analysis was performed comparing the qualitative PET grading, PET/CT grading, SUVmax and SURs. Receiver operator characteristics curves were constructed for this exploratory analysis to compare accuracy between the techniques. The overall discriminatory ability was summarised as the area under the roc curve (AUROC) with 95% confidence interval calculated with 2000 stratified bootstrap replicates. Optimised cut-points were then derived from these ROC curves using the Youdin index. Comparison between the AUROCs was performed using DeLongs test for two correlated ROC curves in *pROC*. To determine the impact of size on diagnostic accuracy, the population was split into tertiles (< 12 mm, 12–16 mm, and > 16 mm), with pairwise comparison of the AUC of the largest nodules with each of the smaller tertiles. To compare the effects of nodule location, we compared nodules in the right and left lower lobes with those in the right and left upper lobes, lingula and right middle lobe. For inter-reader measurement variability assessment between the site and core reads, a one-way random effects model intraclass correlation coefficient was calculated for continuous variables, with Bland-Altman plots also constructed. A square weighted Cohen Kappa was used for ordinal variables and an unweighted Cohen Kappa was calculated for the dichotomous cut points of the qualitative and semi-quantitative PET metrics. *P* < 0.05 was considered statistically significant. Analysis was performed using RStudio (Version 1.1.463, RStudio, Inc.) using the pROC and psych packages [17].

## Results

**Fig. 1:**
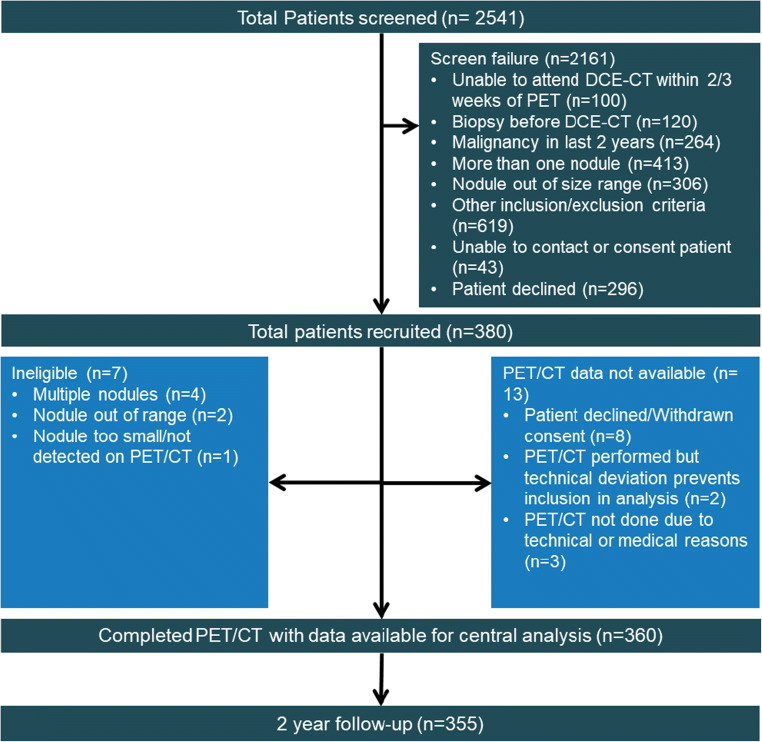
STARD flow diagram of the study recruitment and follow-up. CT—computed tomography; DCE-CT—dynamic contrast enhanced computed tomography; PET—positron emission tomography

Of the 380 participants recruited, 360 completed their PET/CT examinations, with 355 (67.9 ± 9.0 years old, 48% female) completing 2-year follow-up (Fig. 1). Table 1 details the baseline characteristics of the study participants, and the CT findings of their nodule at baseline leading to their recruitment. Fifty-five percent reported as being ex-smokers, with 25% reporting to still be smoking. On the recruitment CT, the nodules were 15.8 ± 5.5 mm on average in diameter (range 8-30 mm), with the majority (241/355, 68%) being described as spiculate (CT grade 3). Of the 355 nodules with 2-year follow-up, 209/355 were malignant (59%).

**Table 1:** Baseline participant demographics, and recruitment CT characteristics

Characteristic	Study population (*n* = 355)
Age, years	67.9 ± 9.0
Sex, female	171 (48%)
Smoking status	
Current	89 (25%)
Ex	195 (55%)
Never	60 (17%)
Respiratory PMH	
COPD	123 (35%)
Asthma	39 (11%)
Pulmonary fibrosis	7 (2%)
TB	10 (3%)
Inhalational exposures	
Asbestos	62 (17%)
Coal	14 (4%)
Silica	4 (1%)
Prior malignancy	46 (13%)
Recruitment CT characteristics	
Nodule location	
Right upper lobe	102 (29%)
Right middle lobe	23 (6%)
Right lower lob	74 (21%)
Left upper lobe	80 (23%)
Lingula	15 (4%)
Left lower lobe	61 (17%)
Size, mm	15.8 ± 5.5
CT grade	
1	14 (4%)
2	67 (19%)
3	241 (68%)
Histology (malignant, *n* = 209)	
Non Small cell lung cancer	147
Adenocarcinoma	112
Squamous cell carcinoma	31
Large cell undifferentiated	2
Not otherwise specified	2
Carcinoid tumour	11
Small cell lung cancer	4
Radiological diagnosis only/SABR	42
Other	5

*COPD*, chronic obstructive pulmonary disease; *CT*, computed tomography; *PMH*, past medical history; *SABR*, sterotactic ablative radiotherapy; *TB*, tuberculosis

**Fig. 2:**
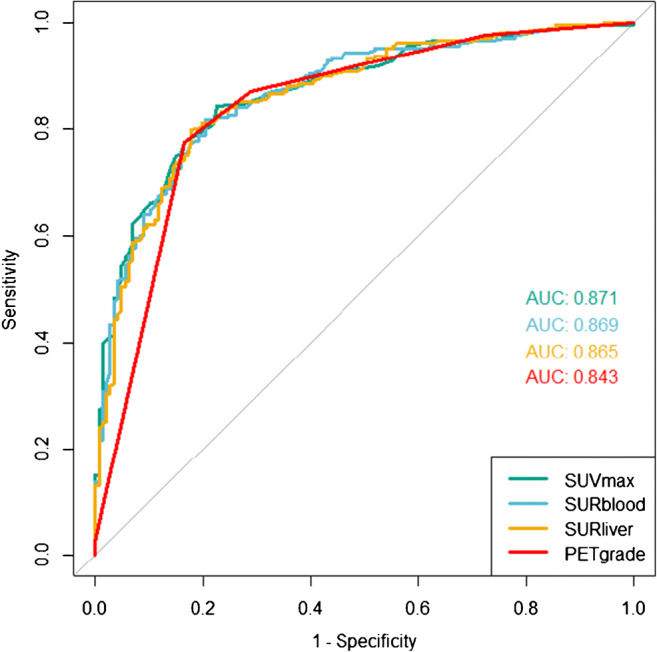
Receiver operator characteristic curve for the diagnosis of malignancy by SUVmax, SUR_BLOOD_, SUR_LIVER_ and PET grade

On PET/CT, malignant nodules were significantly more likely to be spiculate, and have elevated nodule tracer uptake—both absolute and relative to the blood and liver (*p* < 0.001 for all—see Table 2). Of the semi-quantitative markers, SUVmax, SUR_BLOOD_ and SUR_LIVER_ all demonstrated equivalent diagnostic performance (see Fig. 2). The AUCs of the three techniques were as follows: SUVmax 0.87 (95% CI 0.83;0.91); SUR_BLOOD_ 0.87 (95% CI 0.83;0.91, *p* = 0.30 versus SUVmax); SUR_LIVER_ 0.87 (95% CI 0.83;0.90, *p* = 0.09 vs. SUVmax). Considering the techniques as continuous variables, SUVmax, but not SUR_BLOOD_ or SUR_LIVER_, performed significantly better than visual PET-grade (AUC 0.84, 95% CI 0.80; 0.88, *p* = 0.02 vs SUVmax, *p* = 0.06 vs SUR_BLOOD_, and *p* = 0.10 vs SUR_LIVER_). However, when using single-threshold cut-points, a PET uptake of grade 3 showed a similar diagnostic capability to SUVmax≥ 2.5, with a sensitivity/specificity/accuracy of 77.5%/83.6%/80.0% for PET grade and 75.6%/84.2%/79.2% for SUVmax. Both SUR techniques showed similar specificity but poorer sensitivity. In the exploratory analysis, the optimum cut-point shifted in all techniques to a lower threshold with an improvement in sensitivity and a fall in specificity, with new cut points in PET grade of ≥ 2, SUVmax≥ 2.05, SUR_BLOOD_ ≥ 1.26 and SUR_LIVER_ ≥ 0.96. Again, even with these shifted values, PET grade and SUVmax showed similar diagnostic capability. Table 3 details the full sensitivities, specificities, PPV, NPV and accuracies with 95% CIs for both the pre-specified and exploratory analysis.

**Table 2:** PET/CT findings of the nodules in the total cohort, and in those with a final diagnosis of malignant and benign

Characteristic	Benign (*n* = 146)	Malignant (n = 209)	*P* value
Size, mm	12.4 ± 4.5	15.7 ± 5.9	< 0.001
CT grade			< 0.001
0	5 (3%)	2 (1%)	
1	5 (3%)	1 (0.5%)	
2	85 (58%)	48 (23%)	
3	51 (35%)	157 (75%)	
4	0 (0%)	1 (0.5%)	
PET grade			< 0.001
0	40 (27%)	5 (2%)	
1	64 (45%)	22 (11%)	
2	18 (12%)	20 (10%)	
3	24 (16%)	157 (75%)	
4	0 (0%)	5 (2%)	
SUVmax	1.76 ± 1.42	6.39 ± 5.89	< 0.001
SUR_BLOOD_	1.05 ± 0.89	3.60 ± 2.90	< 0.001
SUR_LIVER_	0.75 ± 0.65	2.54 ± 2.12	< 0.001

*CT*, computed tomography; *PET*, positron emission tomography; *SUR*_*BLOOD*_, standardised uptake ratio of nodule compared to mediastinal blood pool uptake; *SUR*_*LIVER*_, standardised uptake ratio of nodule compared to liver uptake; *SUVmax*, standardised uptake value

**Table 3:** Comparison of the diagnostic accuracy using pre-specified and optimised diagnostic threshold cut-points

Technique	Cut-point	Sensitivity (95% CI)	Specificity (95% CI)	Positive predictive value (95% CI)	Negative predictive value (95% CI)	Overall accuracy (95% CI)
Pre-specified						
PET grade	≥ Grade 3	162/209–77.5% (71.2 to 83.0%)	122/146–83.6% (76.5 to 89.2%)	162/186–87.1% (81.4 to 91.6%)	122/169–72.2% (64.8 to 78.8%)	284/355–80.0% (75.4 to 84.0%)
SUVmax	2.5	158/209–75.6% (69.2 to 81.3%)	123/146–84.2% (77.3 to 89.7%)	158/181–87.3% (81.5 to 91.8%)	123/174–70.7% (63.3 to 77.3%)	281/355–79.2% (74.6 to 83.3%)
SUR_BLOOD_	1.56	151/208–72.6% (66.0 to 78.5%)	124/146–84.9% (78.1 to 90.3%)	151/173–87.3% (81.4 to 91.9%)	124/181–68.5% (61.2 to 75.2%)	275/354–77.7% (73.0 to 81.9%)
SUR_LIVER_	1.12	151/208–72.6% (66.0 to 78.5%)	125/146–85.6% (78.9 to 90.9%)	151/172–87.8% (81.9 to 92.3%)	125/182–68.7% (61.4 to 75.3%)	276/354–78.0% (73.3 to 82.2%)
PET/CT grade	*	175/207–84.5% (78.9 to 89.2%)	113/145–77.9% (70.3 to 84.4%)	175/207–84.5% (78.9 to 89.2%)	113/145–77.9% (70.3 to 84.4%)	288/352–81.8% (77.4 to 85.7%)
Exploratory						
PET grade	≥ Grade 2	182/209–87.0% (81.8 to 91.3%)	104/146–71.2% (63.2 to 78.4%)	182/224–81.3% (75.5 to 86.1%)	104/131–79.4% (71.4 to 86.0%)	286/355–80.6% (76.1 to 84.6%)
SUVmax	2.05	176/209–84.2% (78.5 to 88.9%)	113/146–77.4% (69.7 to 83.9%)	176/209–84.2% (78.5 to 88.9%)	113/146–77.4% (69.7 to 83.9%)	290/355–81.4% (77.0 to 85.3%)
SUR_BLOOD_	1.26	170/208–81.7% (75.8 to 86.7%)	116/146–79.5% (72.0 to 85.7%)	170/200–85.0% (79.3 to 89.6%)	116/154–75.3% (67.7 to 81.9%)	286/354–80.8% (76.3 to 84.8%)
SUR_LIVER_	0.96	165/208–79.3% (73.2 to 84.6%)	120/146–82.2% (75.0 to 88.0%)	165/191–86.4% (80.7 to 90.9%)	120/163–73.6% (66.2 to 80.2%)	285/354–80.5% (76.0 to 84.5%)

*CT*, computed tomography; *PET*, positron emission tomography; *SUR*_*BLOOD*_, standardised uptake ratio of nodule compared to mediastinal blood pool uptake; *SUR*_*LIVER*_, standardised uptake ratio of nodule compared to liver uptake; *SUVmax*, standardised uptake value

^*^PET/CT = met one of the following: grade 4 on either PET or CT; Grade 3 on PET and ≥ Grade 2 on CT; ≥ Grade 2 on PET and grade 3 on CT

The pre-specified, combined PET/CT grading provided higher accuracy than the visual grading or the SUVmax pre-specified criteria and provided an equal diagnostic accuracy to the optimised cut points of the exploratory analysis for PET visual and quantitative grading alone (Table 3).

**Fig. 3:**
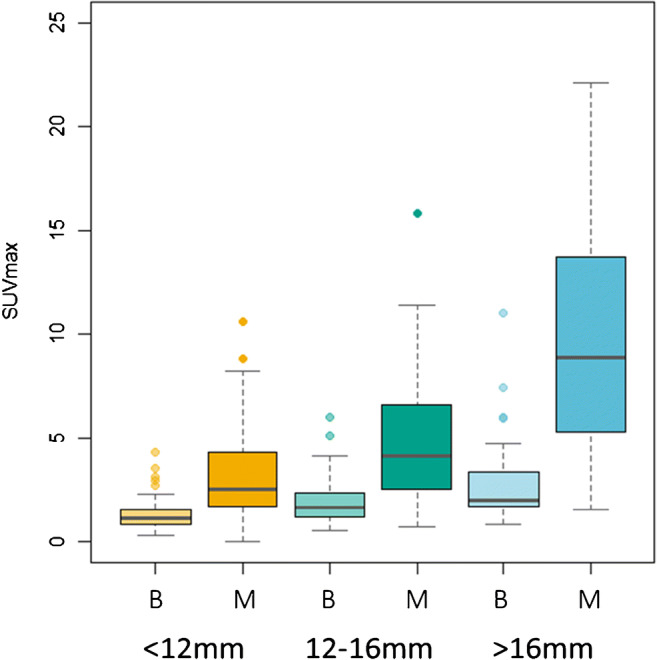
Box and whisker plot of SUVmax by nodule size tertile and malignancy status. B = Benign, M = Malignant. Thick central band = median; upper and lower bound of box = 75th and 25th centiles respectively, whiskers reflecting upper and lower limits, with dots reflecting outliers

As the size of the nodules increased, so too did the SUVmax in both the benign and malignant nodules (see Fig. 3). The size of the nodule had no significant impact on the overall diagnostic accuracy of PET-CT with the AUCs for SUVmax, SURBLOOD, PET grade and PET/CT grade not significantly different for nodules < 12 mm, 12–16 mm or > 16 mm in diameter (*p* > 0.1 for all size group comparisons). However, as the size reduced, there was a fall in sensitivity balanced out by a rise in specificity. This could be counteracted through a shift in the diagnostic threshold for each of the nodules sizes for all three techniques, such that for SUVmax, the optimal cut-point was 1.75 for nodules < 12 mm, 2.55 for nodules 12-16 mm and 3.6 for nodules > 16 mm (see Table 4, and Supplemental Material). In line with this, a PET grade 2 was best used for nodules < 12 mm, while a PET grade of 3 was the optimal cut-point for nodules ≥ 12 mm in size.

**Table 4:** Comparison of area under the receiver operator characteristic curves of SUVmax, SUR_BLOOD_ and PET grade by nodule size

Size	SUVmax		SUR_BLOOD_		PET grade	
	AUC (95% CI)*	Optimal cut-point	AUC (95% CI)*	Optimal cut-point	AUC (95% CI)*	Optimal cut-point
< 12 mm (*n* = 133)	0.84 (0.77; 0.91)	1.75	0.84 (0.76; 0.91)	0.83	0.83 (0.76; 0.90)	≥ Grade 2
12–16 mm (*n* = 122)	0.84 (0.77; 0.91)	2.55	0.83 (0.76; 0.91)	1.76	0.79 (0.71; 0.87)	≥ Grade 3
> 16 mm (*n* = 99)	0.89 (0.82; 0.97)	3.6	0.88 (0.80; 0.97)	2.41	0.83 (0.73; 0.93)	≥ Grade 3

*CT*, computed tomography; *PET*, positron emission tomography; *SUR*_*BLOOD*_, standardised uptake ratio of nodule compared to mediastinal blood pool uptake; *SUR*_*LIVER*_, standardised uptake ratio of nodule compared to liver uptake; *SUVmax*, standardised uptake value

^*^p > 0.1 for all inter-tertile comparison of AUCs

The location of the nodule did not result in a significant difference in the diagnostic performance of SUVmax, with an AUC of 0.82 (95% CI 0.75; 0.89) in the lower lobes versus 0.89 (95% CI 0.85; 0.94) in the mid and upper lobes (*p* = 0.09).

Comparing the Core read with the site read, the SUVmax demonstrated good agreement (ICC—0.92 (95%CI 0.91–0.94)). Bland-Altman plots demonstrated a small but significant bias between the site read and core lab read with wide limits of agreement (mean diff = 0.37 (LOA: − 3.36;4.11, paired *t* test: *p* < 0.001))—see Supplemental Material. Despite this, when considering a threshold of SUVmax> 2.5 as being malignant, there was excellent agreement between the site read and core lab read (Kappa (unweighted): 0.93, *p* < 0.001). The accuracy of SUVmax was the same using both the site and core read (Core AUC 0.87 (95% CI 0.83; 0.91), Site AUC 0.86 (95% CI 0.83; 0.90), *p* = 0.57). As the SUVmax limits of agreement were wider than expected, we further reviewed the steps in the scanning and analysis. Several sites were identified to be using point spread function reconstruction algorithms which can result in an increase in SUVmax of up to 25%. Data submitted to the core lab was all reconstructed using the standard algorithms, but the reporting sites would have had access to both and it is therefore feasible that the values from the point spread function reconstruction would have been recorded in the study case report form. When the 108 cases from these sites were excluded, leaving *n* = 247 in the analysis the mean difference was = − 0.06 (LOA: − 2.29–2.42, paired *t* test: *p* = 0.41), producing significantly improved limits of agreement, and the loss of the bias between the reads. There was excellent agreement between the sites on the visual qualitative grading of the PET uptake (Kappa (with squared weighting): 0.87, *p* < 0.001). The greatest variation in scoring occurred around Grade 2 which is ‘uptake equivalent to the mediastinum’ (Supplemental data). There was fair agreement in the CT visual grading of the nodules (Weighted Kappa (with squared weighting): 0.33, *p* < 0.001), with disagreement between site read and core read most pronounced for Grade 2 lesions (Supplemental data). Despite this, there was good overall agreement on the presence of malignancy on the combined PET/CT grading (Kappa (unweighted): 0.70, *p* < 0.001). There was also no significant difference in the accuracy of the core read versus the site read for either the PET grading (Core: AUC 0.84 (95%CI 0.80; 0.88), Site AUC 0.84 (95% CI 0.80; 0.89), *p* = 0.92) or the PET/CT grading (Core: AUC 0.81 (95%CI 0.77; 0.85), Site AUC 0.82 (95% CI 0.78; 0.86), *p* = 0.70).

## Discussion

This analysis has shown that in a multicentre multivendor environment, SUVmax is highly accurate for the characterisation of nodules, that this is more reproducible between readers, and that normalising this to blood pool or tissue uptake does not improve accuracy. We have found that accuracy remains high in small pulmonary nodules < 12 mm, although diagnostic thresholds need adapting to maintain accuracy both by visual grading and SUVmax.

Single-centre studies have reported on the diagnostic accuracy of PET/CT in the diagnosis of solitary pulmonary nodules. A meta-analysis of 21 such studies found the sensitivity of PET/CT to be 89% (95% CI 87 to 91%) with a specificity of 70% (95% CI 66 to 73%). All reported studies in this meta-analysis used SUVmax for determination of malignancy. However, owing to a lack of standardisation across imaging centres, an estimated 15–20% variability in SUVmax measurements has been reported [18]. As a result, the British Thoracic Society recommend an ordinal visual grading scale for the diagnosis of SPNs, referencing the uptake to the mediastinal blood pool [19]. A single retrospective multicentre study performed by Evangelista et al. found that this visual grading of nodules produced a similar diagnostic accuracy to that of the meta-analysis with a sensitivity of 85.6% (95% CI: 80.4–90.7%), and specificity of 85.7% (95% CI: 80.5–90.9%) [14]. In the current study, we found a lower sensitivity (77.3% (71.0 to 82.8%)) with similar specificity (82.4% (75.3 to 88.2%)) using a visual ordinal grading system. This likely reflects several factors. The current study was prospectively designed and performed, with a high retention rate with only 6% of patients lost to follow-up compared with 29% in the study by Evangelista et al. There was also a high rate of small nodules in our study with 37% of the nodules under 12 mm in maximum diameter.

Despite the overall small size of the pulmonary nodules, we found a good diagnostic accuracy of PET/CT, with this robust to nodule size. While the overall accuracy was unaffected by nodule size, we did however observe a size-dependent effect on nodule uptake, with the magnitude of this effect greater in malignant than benign nodules, consistent with previous studies [20]. As a result of this size-uptake effect, PET/CT specificity increased, and sensitivity fell with falling nodule size. This could however be counterbalanced by altering the thresholds for diagnosis of malignancy. Our current results suggest that, as opposed to using a single cut-point for the diagnosis of pulmonary nodules, that small nodules (< 12 mm) should be considered as malignant if the uptake is equal to the mediastinum on visual inspection, or with an SUVmax ≥ 1.75, while for larger nodules (> 16 mm), the threshold should be increased to an SUVmax ≥ 3.6. This is most likely in part due to partial volume effect. Partial volume correction techniques have been suggested to correct for this; however, a recent meta-analysis found no significant benefits of this with sensitivity gains being more than offset by specificity losses [21]. In comparison, altering diagnostic thresholds by size appears to have salutatory effects on accuracy while also being a tool available to all PET reporters globally, irrespective of hardware or software.

We did not observe a significant impact of nodule location on the accuracy of SUVmax. This is in contradistinction to that of previous studies showing a significantly improved accuracy using respiratory gating [22]. This may reflect the fact that lower lobe nodules include both nodules near the diaphragm where benefits may be maximal as well as nodules in the apical segments where motion will be minimal. Further work is warranted to better understand where maximal benefit may be gained from respiratory gating.

Semi-quantitative analysis of nodules using SUVmax was remarkably robust in the current study. It produced a high diagnostic accuracy and was much more reproducible between readers across multiple sites than was visual ordinal grading. As the study was performed at 16 sites on 25 different scanners covering a range of both models and manufactures, this is an unexpected but important finding. With prior work suggesting inter-scanner variability of absolute SUVmax, there should have been significant noise around the threshold for malignancy. Moreover, normalising this to other tissue uptake should have improved this. Previous work has demonstrated that normalising SUVmax to blood pool or liver uptake to have a high diagnostic accuracy in detection of malignancy in SPNs with AUCs of 0.90 for both liver and blood uptake ratios [14]. We observed a similar high diagnostic accuracy in the current study using this approach, but contrary to expectations, we did not find any ancillary benefit over the use of SUVmax in isolation.

Despite the higher overall accuracy of SUVmax over visual ordinal grading, when using single cut-points such as performed in clinical practice, PET grading was as accurate as SUVmax both in pre-specified and exploratory analysis. Combining the PET grading with the CT grading produced the highest accuracy of all the pre-specified techniques, and equalled those of the exploratory cut points. Given that the latter may be subject to over-fitting to the current data, this suggests that combined PET/CT scoring is the most robust and accurate technique for the analysis of SPNs. This is in agreement with previous studies showing that, while the incremental benefit is small, CT appearances contribute to nodule categorisation in a useful manner [15, 16]. A critical challenge of this combined scoring however is that CT grading appears to be particularly prone to high observer variability, which impacted the diagnosis of malignancy in a large number of cases. This is consistent with previous work showing substantially higher agreement for PET than for CT grading [23]. This variability may have been potentially exacerbated in the current study as the original CT was blinded to the core lab, but not to the site readers. Thus, subtle spiculation, lobulation or heterogeneity of attenuation not evident on the low dose attenuation correction CT may in fact have been determined by the standard CT scan causing this marked upgrade in scoring. However, the equivalent accuracy using core read PET/CT grade and site read PET/CT grade would suggest that any bias introduced by this was small. Lung-RADS, which is a standardised scoring system for nodule assessment in lung cancer screening studies, may be a more reproducible CT scoring system due to its well-defined characteristics in each category [24]. Future studies are warranted to determine if incorporating this within PET/CT scoring further improves accuracy or reproducibility.

Despite the high accuracy and reproducibility of SUVmax, there were significant variations with limits of agreement of − 3.4 to 4.1. This persisted even after removing cases that may have been measured using a different reconstruction algorithm at the reporting and Core lab sites. This degree of variability is in contra-distinction to previous reports where the limits of agreement are typically ± 0.5–0.8 [25, 26]. However, these results were for readers all within a single centre using a single scanner and single post-processing software. In a study by Marom et al. [27] in which 4 readers read 20 scans, they found excellent inter-reader agreement. However, the measures of the 4 readers differed substantially from that documented in the clinical report, with SUV measurements differing by ≥ 25% in 45% of the tumours measured [27].

It is important to note that all centres in the current study underwent central accreditation, quality assurance and adjustment of protocols as necessary to ensure consistent and comparable uptake values by the UK PET Core Lab [13]. The necessity of such work in ensuring applicability of absolute quantitative cut-offs is well appreciated, and similar efforts are underway to improve this globally such as by the EANM/EARL initiative [28, 29].

There are several limitations to the current study. The sample sizes in the nodule size subgroups were relatively small, albeit larger than the majority of existing PET/CT diagnostic accuracy studies. As a result, the confidence intervals around the diagnostic accuracy estimates were larger than in the main study population itself. The proposed sized-based uptake thresholds, particularly for smaller nodules, will require further validation in future studies. The current study had a low rate of diagnosis of infectious diseases as the underlying aetiology of the nodules; thus, the accuracy and thresholds may not be translatable to environments where such conditions are more endemic. Finally, the proposed thresholds only apply to nodule analysis using standard image reconstructions and will require further work to translate into other reconstruction techniques such as point spread function.

In conclusion, we found that in a multicentre multivendor trial, SUVmax was the most accurate and reproducible technique for the diagnosis of solitary pulmonary nodules, and that diagnostic thresholds should be altered according to nodule size.

## Electronic supplementary material

ESM 1 (DOCX 170 kb)

## Data Availability

Individual participant data will be made available, including data dictionaries, for approved data sharing requests. Individual participant data will be shared that underlie the results reported in this article, after de-identification and normalization of information (text, tables, figures, and appendices). The study protocol and statistical analysis plan will also be available. Anonymous data will be available for request from 3 months after publication of the article, to researchers who provide a completed Data Sharing request form that describes a methodologically sound proposal, for the purpose of the approved proposal and if appropriate, signed a Data Sharing Agreement. Data will be shared once all parties have signed relevant data sharing documentation, covering SCTU conditions for sharing and if required, an additional Data Sharing Agreement from Sponsor. Proposals should be directed to ctu@soton.ac.uk.
