# Supplementary material for: Impact of solitary pulmonary nodule size on qualitative and quantitative assessment using 18F-fluorodeoxyglucose PET/CT: the SPUTNIK trial
Source: Eur J Nucl Med Mol Imaging. 2020 Nov 1;48(5):1560–9. doi: 10.1007/s00259-020-05089-y (PMC8113131; doi:10.1007/s00259-020-05089-y)
Supplement: Supplementary file 1 — (DOCX 170 kb) [file 259_2020_5089_MOESM1_ESM.docx]

**Supplemental material:**

**Procedure for ^18^F-FDG-PET/CT**

To maximise consistency of measurements of FDG uptake across multiple sites, we recommend that the ^18^F-FDG-PET scans and examinations should follow the protocol as summarised below:

**Patient Preparation and Scanning:**

- Patient height and weight to be measured and recorded on arrival.
- Patients fast for 6 hours (4 hours for diabetics) before receiving the injection of ^18^F-FDG. While fasting, patients should consume at least two to three 355-mL (12-oz) glasses of water to ensure adequate hydration.
- Diabetics on oral medication should be given a morning appointment, fast for 4 hours and omit their hypoglycaemic medication for the morning.
- Diabetics on insulin should eat and administer insulin as normal then fast for 6 hours prior to appointment. If blood glucose is >11mmol/l, the scan should be rescheduled (INSULIN SHOULD NOT BE GIVEN TO LOWER BLOOD GLUCOSE LEVELS).
- Patients should preferably avoid strenuous exercise for 6 hours before the scan to minimize uptake of the radiotracer ^18^F-FDG in muscles.
- Blood glucose should be measured to determine confirm a concentration of less than 11 mmol/l. Insulin should not be used to adjust the blood glucose at the time of the imaging procedure.
- Intravenous CT contrast media should not be administered prior to the ^18^F-FDG-PET/CT scan. If the DCE-CT scan is to be performed on the same day it should be performed after the ^18^F-FDG-PET/CT scan.
- A separate low dose CT without contrast should also be acquired before the PET acquisition and this scan should be used for attenuation correction of the PET images.
- Before injection of the ^18^F-FDG tracer, patients should urinate to minimize the possibility that they will need to move during the ^18^F-FDG uptake phase.
- The use of all current medications should be noted. Especially those that may affect the uptake or bio- distribution of ^18^F-FDG such as marrow stimulating cytokines or steroids.
- Patients should be placed in a comfortable position, either supine or semi-recumbent, in a dimly lit, quiet room. The room should be kept warm to avoid shivering and other temperature effects that may increase muscular or fat uptake. A large-bore intravenous line (21 gauge or greater) should be placed in an arm or hand vein.
- The injected dose of ^18^F-FDG is dependent on the PET system used and the patient weight (based on a 70Kg individual).
  - For 2D acquisition with a minimum of 5 minutes bed position the target activity is 385MBq (+/-10%) but injected activity must not exceed 400MBq.
  - For 3D acquisitions with an overlap of 25% the minimum activity is 322MBq using 3 minutes per bed position.
  - For 3D acquisition with an overlap of 50% the minimum activity is 240MBq using 2 minutes bed position.
  - For all patients the injected activity must not exceed 400MBq.
  - For patients greater than 90Kg increased scanning time per bed position should be used rather than increase in ^18^F-FDG activity to improve image quality.
- The actual injected ^18^F-FDG activity must be recorded on the PET acquisition form.
- During the ^18^F-FDG uptake the patient should be kept warm to avoid uptake into muscles or brown fat.
- The response scans must be performed at the same time after injection as the baseline scan± 10 minutes.
- The administration of a sedative, such as diazepam, is at the discretion of the clinician.
- Whole-body imaging should ideally begin 60 ± 10 min (mean ± SD) after injection, but this may vary according to local practice.

**Image Acquisition and Reconstruction:**

- Whole-body acquisitions were performed in 3-dimensional mode with attenuation correction.
- The whole-body acquisition should sample from the angle of the jaw to the level of the mid-thigh.
- Images reconstructed with Ordered Subset Expectation Maximization (OSEM) reconstruction with or without Time of Flight (TOF). Where PSF is used, additional reconstructions without PSF should be generated, and used for the study analysis.

**Image Analysis:**

- ^18^F-FDG uptake within SPNs will be quantified as the mean and maximum Standardised Uptake Values (SUVmax) calculated on the basis of both body mass and body surface area.

All scanners underwent baseline accreditation and annual quality assurance testing by the UK PET Core Lab [1]. In addition, the UK PET core lab performed technical checks and image quality review of all studies.

The mean injected activity was 352.6 MBq (range 148.1 to 464.0 MBq). The national diagnostic reference level for 18F-FDG is 400 MBq and it is recommended to use a weight-based protocol of 4.5 MBq/kg with a minimum injected activity dependent on the scanner model and acquisition parameters used (2). The mean injected activity used in the SPUtNIk patients was 4.9 MBq/kg (range 2.5 to 12.2 MBq/kg) and the weight ranged from 30 to 150 kg (mean 76.8 kg). A plot of the injected activity as a function of patient weight is shown in the below Figure.

**Figure.** Injected activity as a function of patient’s weight for all patients

1. Barrington SF, MacKewn JE, Schleyer P, Marsden PK, Mikhaeel NG, Qian W, et al. Establishment of a UK-wide network to facilitate the acquisition of quality assured FDG-PET data for clinical trials in lymphoma. Ann. Oncol. 2011;22:739–45.

**Table S1: List of recruiting sites and PET Centres.**

| **Recruiting Sites (N recruited)** | **PET Centre** | **Scanners** |
| --- | --- | --- |
| Aberdeen (38) | Aberdeen | GE Discovery STE |
|  |  | GE Discovery 710 |
| Brighton, Hastings & Worthing (28) | Brighton | Siemens Biograph 64 |
| Edinburgh (4) | Edinburgh | Siemens Biograph 128 |
| Glasgow (41) | Glasgow | GE Discovery STE |
|  |  | GE Discovery 690 |
| Leicester (12) | Mobile* | 1 x Siemens Biograph 6 |
|  |  | 2 x GE Discovery 710 |
| Leeds (54) | Leeds | GE Discovery 690 |
| Manchester (17) | Christie | GE Discovery STE |
|  | Central Manchester | Siemens Biograph mCT |
|  | Mobile* | GE Discovery 710 |
| Nottingham (24) | Nottingham | GE Discovery 710 |
| Oxford (25) | Oxford | GE Discovery 690 |
| Papworth (74) | Mobile* | 4 x Siemens Biograph 6 |
|  | Addenbrookes | GE Discovery 690 |
| Southampton (21) | Mobile* | 2 x Siemens Biograph 6 |
|  | Portsmouth | Siemens Biograph mCT |
| UCLH (23) | UCLH | GE Discovery VCT |
|  | Royal Free | Siemens Biograph mCT |
| Cheltenham, Weston Park, Worcester (19) | Cheltenham | Philips Gemini GXL |
|  |  | Siemens Biograph 128 |

***8 mobile scanners were used across the sites with the same PET/CT scanner used at more than one recruiting site.**

**Supplemental Table 2:** Sensitivity, specificity and diagnostic accuracy using optimised size-specific cut-points for SUVmax, SURBLOOD and PET grade

| **Nodule Size** | **Cut-point** | **Sensitivity (95% CI%)**  **TP/P** | **Specificity (95% CI%)**  **TN/N** | **Accuracy (95% CI%)**  **TP+TN/P+N** |
| --- | --- | --- | --- | --- |
| **PETgrade** | | | | |
| <12 | ≥ Grade 2 | 42/57 – 74%  (60% to 84%) | 65/76 – 86%  (76% to 93%) | 107/133 – 80%  (73% to 87%) |
| 12-16 | ≥ Grade 3 | 59/75 - 79%  (68% to 87%) | 35/47 - 74%  (60% to 86%) | 94/122 – 77%  (69% to 84%) |
| >16 | ≥ Grade 3 | 72/76 - 95%  (87% to 99%) | 16/23 - 70%  (47% to 87%) | 88/99 - 89 %  (81% to 94%) |
| **SUVmax** | | | | |
| <12 | 1.75 | 42/57 - 74%  (60% to 84%) | 64/76 - 84%  (74% to 92%) | 106/133 – 80%  (72% to 86%) |
| 12-16 | 2.55 | 56/75 - 75%  (63% to 84%) | 37/47 - 79%  (64% to 89%) | 93/122 – 76%  (68% to 83%) |
| >16 | 3.6 | 67/76 - 88%  (79% to 94%) | 18/23 - 78%  (56% to 93%) | 85/99 – 86%  (77% to 92%) |
| **SUR_BLOOD_** | | | | |
| <12 | 0.83 | 49/56 - 88%  (76% to 95%) | 56/76 - 74%  (62% to 83%) | 105/132 – 80%  (72% to 86%) |
| 12-16 | 1.76 | 51/75 - 68%  (56% to 78%) | 41/47 - 87%  (74% to 95%) | 92/122 – 75%  (67% to 83%) |
| >16 | 2.41 | 64/76 - 84%  (74% to 92%) | 19/23 - 83%  (61% to 95%) | 83/99 – 84%  (75% to 90%) |
| **Combined PET/CT grade** | | | | |
| <12 |  | 39/57 – 68%  (55% to 80%) | 67/76 – 88%  (79% to 94%) | 106/133 – 80%  (72% to 86%) |
| 12-16 |  | 65/75 – 87%  (77% to 93%) | 31/46 – 67%  (52% to 80%) | 96/121 – 79%  (71% to 86%) |
| >16 |  | 71/75 – 95%  (87% to 99%) | 15/23 – 65%  (43% to 84%) | 86/98 – 88%  (80% to 94%) |

**Supplemental Table 3:** Confusion matrix comparing the agreement of the recruiting site and the core lab for the PET grade

|  | | | **Core Read** | | | | |
| --- | --- | --- | --- | --- | --- | --- | --- |
|  | | **0** | | **1** | **2** | **3** | **4** |
| **Site read** | **0** | 34 | | 23 | 5 | 1 | 0 |
|  | **1** | 10 | | 51 | 22 | 3 | 0 |
|  | **2** | 0 | | 11 | 8 | 13 | 0 |
|  | **3** | 0 | | 0 | 6 | 165 | 0 |
|  | **4** | 0 | | 0 | 0 | 2 | 0 |

**Supplemental Table 4:** Confusion matrix comparing the agreement of the recruiting site and the core lab for the CT grade

|  | | **Core Read** | | | | |
| --- | --- | --- | --- | --- | --- | --- |
|  | | **0** | **1** | **2** | **3** | **4** |
| **Site read** | **0** | 0 | 0 | 4 | 2 | 0 |
|  | **1** | 1 | 1 | 6 | 12 | 0 |
|  | **2** | 2 | 1 | 65 | 9 | 0 |
|  | **3** | 2 | 4 | 48 | 175 | 0 |
|  | **4** | 0 | 0 | 0 | 0 | 0 |

Figure S1 - Bland-Altman plot comparing SUVmax obtained at the recruiting site versus the core lab across all sites (top) and excluding those with PSF (bottom)


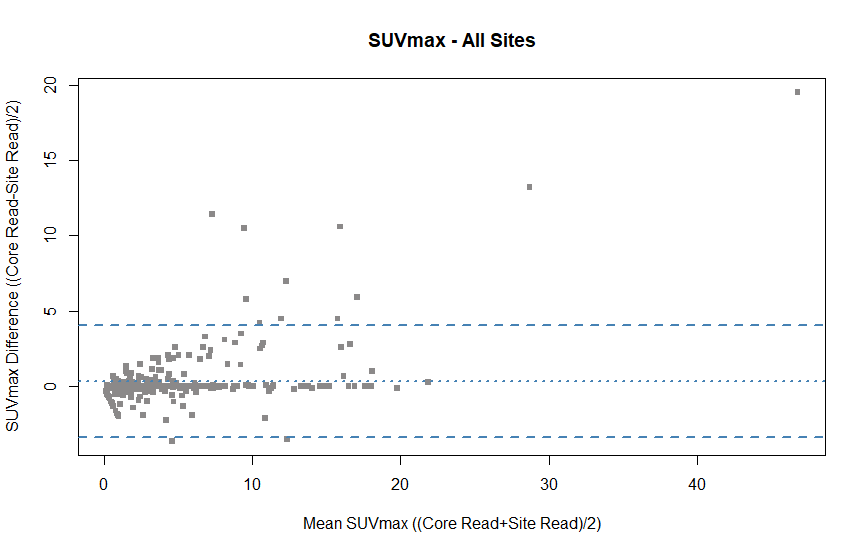


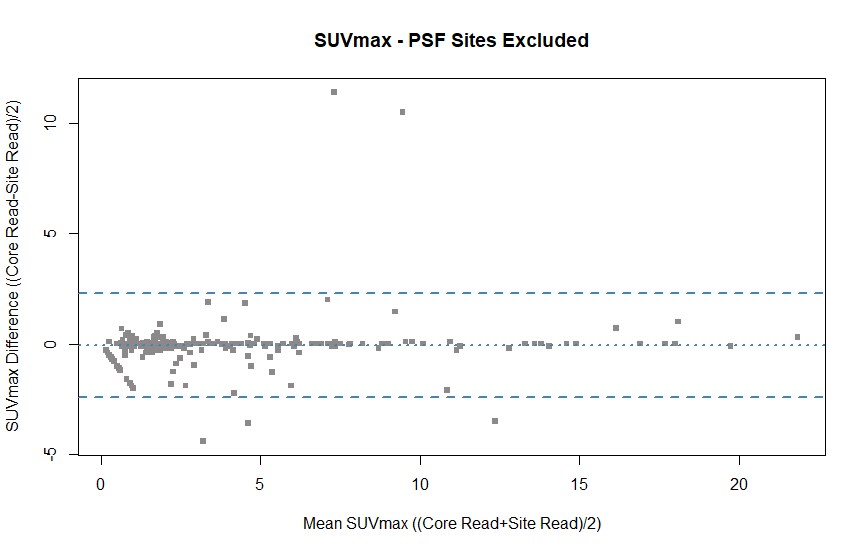


**SPUTNIK investigators:**

Anindo Banerjee, Lucy Brindle, Matthew Callister, Andrew Clegg, Andrew Cook, Kelly Cozens, Philip Crosbie, Sabina Dizdarevic, Rosemary Eaton, Kathrin Eichhorst, Anthony Frew, Fergus Gleeson, Ashley Groves, Sai Han, Jeremy Jones, Osie Kankam, Kavitasagary Karunasaagarar, Lutfi Kurban, Louisa Little, Jackie Madden, Clare McClement, Ken Miles, Patricia Moate, Charles Peebles, Lucy Pike, Fat-Wui Poon, Donald Sinclair, Andrew Shah, Luke Vale, Steve George, Richard Riley, Andrea Lodge, John Buscombe, Theresa Green, Amanda Stone, Neal Navani, Robert Shortman, Gabriella Azzopardi, Sarah Doffman, Janice Bush, Jane Lyttle, Kenneth Jacob, Joris van der Horst, Joseph Sarvesvaran, Barbara McLaren, Lesley Gomersall, Ravi Sharma, Kathleen Collie, Steve O'Hickey, Jayne Tyler, Sue King, John O'Brien, Rajiv Srivastava, Hugh Lloyd-Jones, Sandra Beech, Andrew Scarsbrook, Victoria Ashford-Turner, Elaine Smith, Susan Mbale, Nick Adams, and Gail Pottinger
